# Ocean Acidification at High Latitudes: Potential Effects on Functioning of the Antarctic Bivalve *Laternula elliptica*

**DOI:** 10.1371/journal.pone.0016069

**Published:** 2011-01-05

**Authors:** Vonda Cummings, Judi Hewitt, Anthony Van Rooyen, Kim Currie, Samuel Beard, Simon Thrush, Joanna Norkko, Neill Barr, Philip Heath, N. Jane Halliday, Richard Sedcole, Antony Gomez, Christina McGraw, Victoria Metcalf

**Affiliations:** 1 National Institute of Water and Atmospheric Research, Wellington, New Zealand; 2 National Institute of Water and Atmospheric Research, Hillcrest, New Zealand; 3 Department of Wine, Food and Molecular Biosciences, Lincoln University, Lincoln, New Zealand; 4 National Institute of Water and Atmospheric Research, Dunedin, New Zealand; 5 Environmental and Marine Biology, Åbo Akademi University, Åbo, Finland; 6 Mahanga Bay Aquaculture Facility, National Institute of Water and Atmospheric Research, Wellington, New Zealand; 7 Chemistry Department, University of Otago, Dunedin, New Zealand; Argonne National Laboratory, United States of America

## Abstract

Ocean acidification is a well recognised threat to marine ecosystems. High latitude regions are predicted to be particularly affected due to cold waters and naturally low carbonate saturation levels. This is of concern for organisms utilising calcium carbonate (CaCO_3_) to generate shells or skeletons. Studies of potential effects of future levels of pCO_2_ on high latitude calcifiers are at present limited, and there is little understanding of their potential to acclimate to these changes. We describe a laboratory experiment to compare physiological and metabolic responses of a key benthic bivalve, *Laternula elliptica*, at pCO_2_ levels of their natural environment (430 µatm, pH 7.99; based on field measurements) with those predicted for 2100 (735 µatm, pH 7.78) and glacial levels (187 µatm, pH 8.32). Adult *L. elliptica* basal metabolism (oxygen consumption rates) and heat shock protein *HSP70* gene expression levels increased in response both to lowering and elevation of pH. Expression of chitin synthase (*CHS*), a key enzyme involved in synthesis of bivalve shells, was significantly up-regulated in individuals at pH 7.78, indicating *L. elliptica* were working harder to calcify in seawater undersaturated in aragonite (Ω_Ar_ = 0.71), the CaCO_3_ polymorph of which their shells are comprised. The different response variables were influenced by pH in differing ways, highlighting the importance of assessing a variety of factors to determine the likely impact of pH change. In combination, the results indicate a negative effect of ocean acidification on whole-organism functioning of *L. elliptica* over relatively short terms (weeks-months) that may be energetically difficult to maintain over longer time periods. Importantly, however, the observed changes in *L. elliptica CHS* gene expression provides evidence for biological control over the shell formation process, which may enable some degree of adaptation or acclimation to future ocean acidification scenarios.

## Introduction

The ocean and the land are ‘sinks’ for the excess atmospheric CO_2_ produced by burning of fossil fuels, deforestation and land use changes. Twenty five to thirty percent of the total anthropogenic CO_2_ emissions produced since the industrial revolution have been absorbed by the oceans [1]. This excess CO_2_ dissolves in the surface ocean, causing increased hydrogen ion (H^+^) concentrations and decreased carbonate ion (CO_3_^2−^) concentrations in seawater [2]. The result is a decline in ocean pH and calcium carbonate (CaCO_3_) saturation states. Through this process, known as ‘ocean acidification’, pH has already dropped by 0.1 pH units (an increase in H+ concentration of almost 30%) since the 1800s, and the rate of change is predicted to increase considerably, with a further decline of 0.2–0.5 pH units expected by 2100 [3], [4].

While the implication of ocean acidification to the chemistry of the open ocean is reasonably well understood, the ecological implications for marine fauna and flora are harder to predict [5], [6]. The taxonomic groups considered most susceptible to ocean acidification are calcifying organisms with CaCO_3_ skeletons and shells, such as corals, coralline algae, coccolithophores and molluscs. This is due to the predicted reduced availability of the CO_3_^2−^ ions they require for precipitation of CaCO_3_ [5], [7]. The degree of susceptibility of these calcifiers to ocean acidification is thought to be influenced by their mineralogy (but see [8], [9]). For example, skeletons made of the CaCO_3_ polymorph aragonite are generally more soluble than those comprised of calcite, but calcite skeletons containing high proportions of magnesium may be even more soluble than aragonite [10]. Organisms without CaCO_3_ skeletons or shells have also been shown to be affected through disruption of their acid-base balance and respiration (e.g., [11]–[13]). Experiments to date have shown that species’ responses are variable, due in part to differences in functional responses and environmental conditions (e.g., [14], [15]). Importantly too, not all species, nor all life stages of a particular species, have responded negatively to pCO_2_ conditions predicted for the future (e.g., [16], [17]).

Especially fast rates of change are expected in the Southern Ocean due to its cold water temperatures (and thus higher solubility of CaCO_3_, [18]) and CO_3_^2−^ saturation levels that already are lower than temperate regions. As early as 2030 during winter months, the Southern Ocean is predicted to become undersaturated in aragonite [19], [20]; a situation that has probably not occurred in at least the last 400,000 years [4]. Studies of potential effects of future levels of pCO_2_ on high latitude calcifying organisms are at present limited. Those conducted to date have shown reduced calcification and higher dissolution rates of shells of live pteropods [4], [21] and dissolution of bivalve, gastropod and brachiopod shells [9], and have documented a 30% decline in shell weights of foraminifera since the late 1800s [22]. In contrast, fertilisation, early embryogenesis and larval development of high Antarctic sea urchins were unaffected except at pH below 7.3 (i.e., pCO_2_ predicted for 2300; [23], [24]), and similar results were noted for fertilisation and early development of nemerteans [24].

*Laternula elliptica* is a large, infaunal, suspension-feeding bivalve with a circum-Antarctic distribution. It has a wide depth range (1–460 m), but is particularly common in shallow waters where it is routinely found at densities of 10's of individuals m^−2^ [25] and often much higher (>170 ind. m^−2^; [26], authors' unpubl. obs). In McMurdo Sound, Ross Sea, *L. elliptica* occurs in a variety of habitat types, ranging from homogeneous soft sediments to coarse pebbly sand deposits between rocks (authors' unpubl. obs). *L. elliptica* is a key species in Antarctic coastal benthic ecosystems through its influence on benthic-pelagic coupling [25] and has a shell comprised purely of aragonite [27]. In a laboratory study, [9] demonstrated significant dissolution of adult *L. elliptica* shells after 28 days exposure to pH 7.4. However, as their study was conducted on empty valves only, the biological response by the *L. elliptica* and their potential to compensate for this dissolution remains unknown.

Here we describe a laboratory experiment designed to assess the effect of a change in ocean pH on the functioning of adult *Laternula elliptica* from the Ross Sea, Antarctica. We compared *L. elliptica*'*s* biological response at current Antarctic pH (7.99) to that predicted for the high Antarctic in the following decades (7.78; [4], [19]),and to a considerably elevated, ‘glacial’ pH (8.32). A suite of response variables, chosen to incorporate a range of molecular level (gene function, of stress and growth proteins) and whole organism responses (respiration and physiological condition) important in the functioning of this bivalve, were assessed. Specifically, we monitored effects on *L. elliptica* (i) shell, via measurement of chitin synthase (*CHS*) gene expression, a key enzyme involved in the shell formation process [28], and changes in shell weight; (ii) metabolism and growth, via measurement of oxygen consumption [29], physiological condition [30] and protein synthesis (i.e., total RNA, [31]); and (iii) stress, via measurement of heat shock proteins (*HSP*, specifically *HSP70* gene expression, [32]). By examining this range of interlinked variables we were able to build a more comprehensive picture of the likely effect of ocean acidification on the functioning of this key Antarctic bivalve.

## Methods

*Laternula elliptica* were collected from 20 m depth at Granite Harbour, Ross Sea, Antarctica by SCUBA divers on 15 November 2008. Their habitat consisted of loosely compacted coarse, sandy sediments, interspersed with pebbles and cobbles, and with very low CaCO_3_ content (<1%). Live *L. elliptica* (average 72.8 mm shell length (SL), range 54.2–87.9 mm) were transported to Wellington, New Zealand on 20 November 2008, where they were immediately transferred to a laboratory facility and held in running seawater. Seawater was sourced from the adjacent Wellington Harbour (WH) and was used in a single pass flow through system, filtered on a 0.1 µm filter and chilled to the experimental temperature of −1.76°C (± 0.0008 SE; range −1.93 to −1.60°C). WH seawater was of similar salinity to that at the Granite Harbour collection site (34.1 and 34.6, respectively).

### pH/pCO_2_ manipulation

Three pH treatment levels were chosen for the experiment. These included an ‘Antarctic control’ (pH 7.99), a lowered pH (pH 7.78) which represents that predicted for 2100 by [4], and an elevated pH (pH 8.32) which last occurred in the Antarctic 20,000 years ago [33]. The Antarctic control pH treatment level was chosen based on measurements of water samples collected at the study site. The calculated pCO_2_ equivalents for the elevated, Antarctic control, and lowered pH treatments are 187, 430, and 735 µatm, respectively (see Table 1).

**Table 1:** Chemical characteristics of seawater from the experimental treatments, field measurements made at two McMurdo Sound locations, and relevant polar studies.

	ExperimentElevated pH pH 8.316±0.001	ExperimentAntarctic control pH 7.993±0.002	ExperimentLowered pH pH 7.775±0.002	Field Granite Harbour pH 8.024	Field New Harbour pH 7.996±0.007	Cape Armitage pH 8.0 [23]	Arctic Fjord pH 8.12 [21]	Arctic Fjord pH 7.78 [21]	Artificial seawater pH 7.4 [9]	Artificial seawater pH 8.2 [9]
pCO_2_ ppm (c)	186.5+0.3	429.5+0.8	734.6+0.9	410	439.6±6.9	nr	320	765	nr	nr
A_T_ µmol kg^−1^	2253.2±3.9	2256.5±3.9	2258.3±2.8	2338.6	2329±2.8	2336 (c)	2312	2295	2558	2678
C_T_ µmol kg^−1^	2043.7±3.7 (c)	2169.4±3.9 (c)	2235.0±2.8 (c)	2238.2	2238.1±2.3	nr	2148 (c)	2237 (c)	nr	nr
Ω_Ar_ (c)	2.178±0.004	1.133±0.002	0.710±0.001	1.261	1.18±0.02	1.03	1.81	1.0	0.47	2.66
[CO_3_^2−^] µmol kg^−1^ (c)	144.0±0.3	74.9±0.1	47.0±0.1	83.5	78.4±1.2	nr	nr	nr	nr	nr
Temperature (°C)	−1.76±0.001	−1.76±0.001	−1.76±0.001	−1.92	−1.92	−1.9	2.2	5.0	4.0	4.0
Salinity (ppt)	34.1	34.1	34.1	34.6	34.6	34.6	34.9	34.8	35	35

Values from this study are mean ± standard errors. A_T_ = average total alkalinity, C_T_ = total dissolved CO_2_, Ω_Ar_ = aragonite saturation index. (c) indicates calculated values, all other values are measured, nr = not reported. pH on the total hydrogen scale except for [23] ( =  NBS scale) and [9] ( =  not specified).

The pH of the chilled seawater was manipulated by bubbling food-grade 100% CO_2_ gas into a 60 litre tank of WH seawater to reduce the pH to 7.6 pH units. This low pH water was then mixed with WH seawater in additional 60 litre ‘header’ tanks to produce the Antarctic control and lowered pH treatments, respectively. The quantity of low pH water pumped to the header tanks was controlled via a Proportional Integrative Derivative feedback loop from industrial pH controllers (ATI Q 45P, Analytical Technology Inc. USA). The controllers measured the header tank pH through high quality glass pH electrodes (Hamilton, Switzerland) (Liq-Glass rated to -10°C to +100°C) and were temperature compensated using PT100 resistance thermometers (Servotech, New Zealand). The pH electrodes were calibrated regularly. The more accurate pH measurements derived from spectrophotometric readings (described below) were used to correct for any drift in the pH control system between calibrations. The elevated pH treatment was generated by chilling WH seawater to experimental temperature. Water from the header tanks was gravity-fed to replicate tanks (each 20 l) at a rate of 140 ml min^−1^. There were six replicates of each of the three experimental treatments.

### Experimental setup

*Laternula elliptica* were individually numbered using permanent marker pen, their SL measured using electronic callipers, and 8 individuals were added to each replicate tank. Each *L. elliptica* was placed into tanks in a small mesh basket, which ensured they were held in a lifelike orientation (siphons uppermost). The tanks did not contain sediment.

Seawater pH in the replicate tanks was initially set at chilled WH levels (8.32). The pH was then gradually lowered to the target levels over the following 24 h, reaching these on 27 November 2008 (Day 0). *L. elliptica* were fed twice per week with a commercial algae mix (Shellfish diet, Reed Mariculture, US).

### Chemical characteristics of seawater

Water samples for alkalinity (A_T_) were collected from the header tanks throughout the experiment, on December 4^th^ and 18^th^ 2008, and March 25^th^ 2009 (n = 13, 14 and 13 for pH 8.32, 7.99 and 7.78, respectively), and preserved using HgCl_2_. A_T_ was determined using a closed cell potentiometric titration method [34]. The accuracy of this method is estimated to be 1.5 µmol kg^−1^, based on the analyses of Certified Reference Material supplied by Andrew Dickson. pCO_2_, aragonite saturation (Ω_Ar_), carbonate ion concentrations ([CO_3_^2−^]) and dissolved inorganic carbon (C_T_) were calculated from the measured A_T_ and pH at the average experimental water temperature and salinity using the refitted [35] equilibrium constants [36]. Salinity of WH seawater was measured daily as conductivity with Endress Hausser CLS30 - DIC4A probes.

For the first 38 days of the experiment (from 26/11/08 to 4/1/09), pH was measured manually twice daily using a pH electrode (WTW Multi 340i with sensorex glass probe). From 20/12/08 onwards, pH was measured spectrophotometrically using an automated system (see [37] for details) which sampled the header tanks hourly and the replicate tanks every 12 h. The dual measurements made during the 16 day overlapping time period allowed us to correct the electrode-based measurements using the more accurate spectrophotometric measurements, and thus attain a continuous record for the experiment. For spectrophotometric pH measurements, seawater from the tank being measured was drawn into a syringe pump (Kloehn model 55022, fitted with a 50 ml syringe), then mixed with an aliquot of thymol blue dye (mixing ratio 49:1). The dye-seawater mixture was pumped into a spectrometer (Ocean Optics USB4000) fitted with a 1 cm path length quartz cuvette. The spectral data were combined with reference data (no dye) to determine the absorbance at 435, 596 and 735 nm. The syringe pump and spectrophotometer assembly were located in a box which was temperature controlled at 4.2°C, and the temperature of the cuvette was recorded along with the absorbance data. The water sampling, pH measurement and data logging was automated, and controlled by a LabView programme. pH on the total hydrogen scale was calculated from the measured absorbances and the thymol blue dye parameters (pK2, e1, e2, and e3) at the cuvette temperature and a salinity of 34.1, using the algorithms of [38]. The pH at experimental water temperature was then calculated from the pH measured at 4.2°C using the A_T_ and the [35] equilibrium constants as refitted by [36].

### *Laternula elliptica* sampling

*L. elliptica* were sacrificed from each replicate tank on Days 0, 21 and 120 (27 November, 18 December 2008 and 28 March 2009, respectively) for determination of physiological and/or biochemical parameters. On Day 21, individuals were assessed for gene expression characteristics indicative of stress (i.e., heat shock protein, *HSP70*) and calcification activity (*CHS*). On Days 21 and 120, assessments of overall protein synthesis potential (i.e., total RNA content) were made. Also on Day 120, the individuals used for protein synthesis potential were first used to determine oxygen consumption rates as a proxy for energy use. On Days 0 and 120, physiological condition of the individuals was determined. Different individuals were assessed for physiological and biochemical parameters, respectively, on any one sampling date. Processing of *L. elliptica* for each of these measured is detailed below.

1. Molecular analyses

Mantle and adductor tissue were carefully dissected from each individual, snap frozen in liquid nitrogen and stored at −80°C prior to analysis for (a) *HSP70* and *CHS* gene expression levels and (b) total RNA quantification, respectively, as described below.

*A. Gene expression analysis*

The messenger RNA (mRNA) levels in mantle tissue of *L. elliptica* target genes (*CHS*, *HSP70*) were measured by reverse transcription quantitative polymerase chain reaction (RT-qPCR), a highly sensitive method which provides a measure of the relative expression levels of the target mRNA versus a control or reference gene (such as β-actin). Mantle tissue was chosen for analysis as: 1) this is the site of synthesis of calcification proteins, such as our target protein, CHS; and 2) highest expression of HSPs and constitutive expression of *HSP70* occurs in this tissue in *L. elliptica* [39]. Expression analysis was achieved by first isolating total RNA, followed by the generation of cDNA, which was subsequently used in RT-qPCR. As the *CHS* gene sequence has not previously been determined from *L. elliptica*, this first necessitated PCR amplification of a partial *CHS* cDNA fragment using degenerate primers followed by cloning and sequencing prior to the development of a RT-qPCR assay for this gene (ii below).

Total RNA was extracted from 100 mg of *L. elliptica* mantle tissue using TRIzol reagent (Invitrogen Co, Grand Island, NY, USA) and resuspended in DEPC-treated water. The concentration of total RNA was determined by measuring ultraviolet absorbance at 260 nm. RNA purity was checked by determining the *A*260/*A*280 ratio, and RNA integrity was checked by agarose gel electrophoresis.

RNA samples were treated with Deoxyribonuclease I (Sigma-Aldrich) to remove any contaminating genomic DNA. Single-strand cDNA was reverse transcribed from 100 ng total RNA in a final volume of 10 µl using random primers and MMLV RT as per the SuperScript VILO cDNA synthesis kit (Invitrogen). Reactions were incubated at 25°C for 10 min, then at 42°C for 90 min, and terminated by heating at 85°C for 5 min. cDNA was stored at −20°C until required for cloning or PCR.

Degenerate primers for amplifying a circa 800 bp cDNA fragment of the *L. elliptica CHS* gene were designed on the basis of known molluscan *CHS* cDNA sequences (Table 2). PCR was performed using 10–100 ng of cDNA as a template in PCR buffer containing 3 mM MgCl_2_, 0.2 mM dNTPs, 0.4 µM of each primer and 1 unit of Platinum Taq Polymerase (Invitrogen) in a total volume of 10 µl. The thermal cycling parameters used were 95°C denaturation for 2 min, followed by 35 amplification cycles of 95°C for 30 sec, 55°C for 30 sec, 72°C for 2 min, and a final extension at 72°C for 10 min. The PCR products were gel-purified and sub-cloned into pCR4 TOPO TA vector (Invitrogen), and sequenced on an ABI Prism 3130 sequencer from both the 5′ and 3′ ends using the ABI PRISM dye terminator cycle sequencing ready reaction kit (Applied Biosystems, Foster City, CA, USA). Complete clone insert sequences were identified using the blast or blastx search programs within the National Center for Biotechnology Information, in order to confirm their identity as *CHS*.

**Table 2:** Oligonucleotide primers used for cloning and qPCR of target genes from *Laternula elliptica.*

Gene Target	Oligo	Primer DNA sequence (5′-3′)
**Chitin Synthase**		
Cloning primer	*CHS*3F	TGyGCnACnATGTGGCAyGArAC
	*CHS*3R	GCyTTyTGnArCCArTGnCC
qPCR primer	LE q*CHS*F	TGTCCGCTCCTATCAAAACC
	LE q*CHS*R	GGCCTTATCTCCTTCCTTGG
***HSP70*** *		
qPCR primer	HspF	AGATGAGGCTGTTGCATACG
	HspR	GGTGACGTCAAGAAGAAGCA
β-actin*		
qPCR primer	ACTF	GGTCGTACCACAGGTATTGT
	ACTR	CATCAGGTAGTCGGTCAAAT

Primers from [40]; *HSP70* Genbank accession number EF198332, β-actin Genbank accession number EF198331

PCR amplifications of target (*CHS*, *HSP70*) and reference (β-actin) genes were performed in 25 µl reactions containing: cDNA generated from 200 pg of original RNA template (10 µl of 1∶500 diluted cDNA generated from 100 ng of original RNA template), 0.2 µM of each gene specific primer (GSP, Table 2), 10 nM fluorescein, 0.2 mM dNTPs, 3 mM MgCl_2_, 0.33x Sybr Green, 0.15% Triton X-100, 1 U of Platinum Taq polymerase (Invitrogen) and 1X PCR buffer (Invitrogen (20 mM Tris-HCl (pH 8.4), 50 mM KCl)). Note that primers have been previously developed for β-actin and *HSP70* qRT-PCR [39]. Amplification was performed and monitored using the Icycler IQ real-time PCR detection system (Biorad, Hercules, CA, USA) with the following thermal cycle protocol: initial denaturation and enzyme activation at 95°C for 2 min, 40 amplification cycles of 94°C for 15 sec, 58°C for 15 sec and 72°C for 15 sec, followed by a melt curve analysis. Normalisation was performed by collecting dynamic well factors using the fluorescein background signal detected during the first few PCR cycles, in order to compensate for any system or pipetting non-uniformity and optimise fluorescent data quality and analysis.

Each template was analysed in triplicate. A four point standard curve for each primer pair being assayed, containing 10-fold dilutions of cDNA prepared from stock cDNA covering five orders of magnitude (1, 0.1, 0.001, 0.0001, and also encompassing the target nucleic acid interval), was included in triplicate on each 96-well PCR plate.

Data were collected as quantification cycle (C_q_, formerly cycle threshold (C_t_)) values using the iCycler iQ Optical System Software Version 3.1. The C_q_ values were normalised to sample quantities using the standard curve method for relative quantification. The primer concentrations (0.2 µM) were empirically determined based on lowest C_q_ values and highest efficiencies. The β-actin gene of *L. elliptica* was used as a reference gene. Data were normalised against the expression of β-actin (expressed as a ratio) to compensate for any differences in loading or reverse transcriptase efficiency. β-actin, a widely used reference gene in qPCR analysis, has previously been used for qPCR analyses in *L. elliptica* [40], and 1-way ANOVA demonstrated this gene did not vary across each time point in response to our pH treatments (data not shown).

*B. Total RNA content*

Total RNA on Days 21 and 120 was determined by pulverising freeze dried adductor tissue in a glass mortar and subsampling for RNA quantification. Total RNA was extracted using the TRIzol Reagent (Invitrogen # 15596-018; [41]), and quantified spectrophotometrically. Approximately 15 mg of tissue was used for the analyses and an additional ethanol wash was added to the manufacturer's protocol to ensure samples of satisfactory purity. Total RNA was quantified by reading the absorption against 0.5% SDS at 260 and 280 nm with a spectrophotometer using quartz cells (1.4 ml volume). In addition, absorption spectra were obtained (200–320 nm) as an indicator of sample purity. To standardise RNA content between individuals of different sizes, we normalised results to the average SL of the *L. elliptica* used in the experiment, 73 mm, using the following formula:

Total RNA ind A_adj_ = [RNA ind A (µg mg^−1^)/SL ind A (mm)]×73

Results are expressed as µg RNA mg^−1^ tissue dry weight.

2. Oxygen consumption

Respiration rates of *L. elliptica* were determined on Day 120. Individuals were placed in closed 600 ml chambers in their equivalent treatment water, with continuous, gentle propeller-driven mixing, after first brushing and rinsing to remove microflora. Chambers were placed in a water bath and temperatures maintained at −1.4°C (range = −1.3 to −1.6°C). After 50 min the chambers were sealed and oxygen consumption measurements were made over 184 min. Fifty min was considered to be an appropriate settling period to eliminate any effects of handling stress, based on trial experiments that we had conducted and other studies (e.g., [42], [43]). Measurements were made using hand held probes (YSI55, YSI550A and WTW Multi 340i with CellOx probe), which were first calibrated using fully saturated (100%) water and then sulfite (H_2_S) water (0%). Dissolved oxygen saturation in each chamber was not allowed to fall below 70%. O_2_ consumption was recorded every 2 min as % saturation and every 5 min as mg l^−1^. These values were later adjusted for the volume of the animal and for consumption rates recorded in chambers without animals.

To convert O_2_ consumption values to per unit ash free dry weight (AFDW; methods provided below), we derived the following relationship between *L. elliptica* AFDW and wet tissue weight:

AFDW = 0.37014 wet weight^0.55134^ ; R^2^ = 0.63

As there were no differences in this relationship between animals from different treatments (standard errors of all parameter estimates overlap for all treatments) we considered this to be a good estimator of AFDW for the O_2_ consumption animals.

3. Physiological condition

On Days 0 and 120, *L. elliptica* from each experimental replicate were dissected to remove flesh, the flesh wet weight determined, and the flesh and shell oven dried (60°C) and air dried, respectively, to constant weight (hereafter ‘FW’ and ‘SW’, respectively). The dry flesh was then ashed in a muffle furnace at 470°C for 24 h to determine AFDW (AFDW = FW- ashed weight). Two physiological condition indices were calculated: the ratio of FW to SL (CI_FW:SL_), and the ratio of SW to SL (CI_SW:SL_). We also examined the change in each condition index over the 120 day experiment (Day 120 – Day 0).

### Statistical analyses

A 2-way ANOVA with Day as a random factor and Treatment as a fixed factor was initially conducted to confirm that SL of the animals did not vary between treatments.

Where simple monotonic relationships between each response variable and pH were indicated, the significance of the relationship was tested by correlation analysis (either Pearson's or Spearman's) due to the well known insensitivity of ANOVA to monotonic gradients in responses [44]–[46]. For response variables exhibiting non-monotonic relationships, 1-way ANOVAs were used to test for differences between treatments, after determining whether normality and homogeneity of variances assumptions were met. When this was not the case and transformations did not help, a Kruskal-Wallis test was used.

### Antarctic field conditions

Background information on the water chemistry in McMurdo Sound was determined from day time water samples and longer term instrument deployments. On 10 November 2008 water samples were collected for C_T_ and A_T_ analyses from 3 m below the surface at the Granite Harbour collection site and later analysed to determine the Antarctic control treatment pH level. Near bottom (18 m deep) water samples for C_T_ and A_T_ analyses were collected from New Harbour on seven occasions in the following year (6^th^ and 8–14 November 2009). All samples were immediately preserved with HgCl_2_. A_T_ was analysed as described above, and C_T_ was determined using coulometric measurement of the CO_2_ evolved from an acidified sample [34]. In situ pH, pCO_2_, Ω_Ar_ and [CO_3_^2−^] were calculated from the measured values using the refitted [35] equilibrium constants [36]. Information on temperature and salinity required for these calculations was obtained at both sites from Seabird Electronics SBE-37 microcat deployments.

## Results

### Experimental conditions

The chemical characteristics of our experimental treatments and Antarctic field sites are given in Table 1. Also provided for comparison are characteristics of water reported in other relevant polar studies from the literature. These values show good agreement with our experimental conditions. Of interest are the high pCO_2_ levels of our McMurdo Sound water samples (410 and 440 µatm at Granite Harbour and New Harbour, respectively).

### *Laternula elliptica* response

There was no mortality over the 120 day experiment, and *L. elliptica* appeared to behave normally, extending siphons and feeding. There was also no measurable increase in SL of these adult *L. elliptica* over the experiment. Sizes of individuals did not differ significantly between treatments (p>0.05).

1. Molecular analyses

*A. HSP70, heat shock protein gene expression*

**Figure 1:**
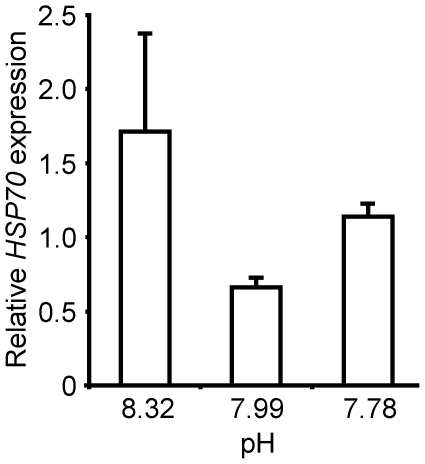
mRNA expression of *HSP70* in *Laternula elliptica* mantle tissue after 21 days at experimental pH. Levels shown are means (+ SE) relative to β-actin (expressed as a ratio, fold induction).

*L. elliptica* mRNA expression levels of *HSP70* showed a curvilinear response to seawater pH, with lowest levels in the Antarctic control treatment after 21 days. There was considerably higher variability in *HSP70* levels of individuals from the elevated pH treatment than in those of the other pH treatments (Figure 1). There was no significant correlation between pH and *HSP70* gene expression relative to reference gene β-actin expression. However, *HSP70* expression showed some indication of differences between treatments (Kruskal-Wallis Chi-Square = 5.3450, p = 0.0691), with the Antarctic control differing from both the elevated and lowered pH treatments (unadjusted p-values were 0.0451 and 0.0039, respectively).

*B. CHS cloning, sequence determination and gene expression*

**Figure 2:**
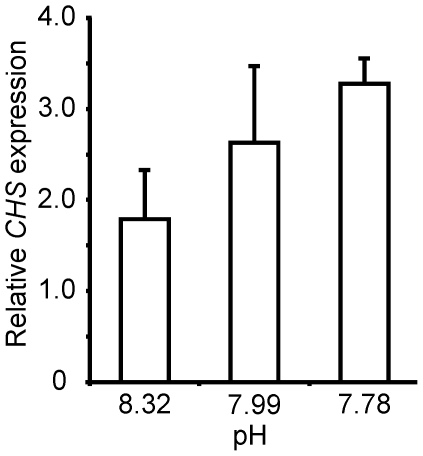
mRNA expression of *CHS* in *Laternula elliptica* mantle tissue after 21 days at experimental pH. Levels shown are means (+ SE) relative to β-actin (expressed as a ratio, fold induction).

There was a significant negative correlation between mRNA expression levels of *CHS* relative to β-actin reference gene expression in *L. elliptica* and pH (log_10_ transformed) on Day 21 of the experiment (Pearson's R −0.61, p = 0.0060, n = 18), with an increase in *CHS* with decreasing pH (Figure 2).

The *L. elliptica CHS* partial cDNA sequence of 953 nucleotides (Genbank accession number HQ186262) is provided in Figure S1 overlaid with the deduced protein sequence of 317 amino acids. Highly conserved motif regions 1–4 found in many family 2 glycosyltransferases (GTF2) enzymes, including CHS, are indicated. BLAST analysis showed that the *L. elliptica CHS* nucleotide sequence gene shares homology with other known *CHS* genes, indicating that the cloned gene encodes CHS protein (Figure S2). A multiple sequence alignment of the deduced protein sequence of *L. elliptica* CHS with chitin synthases of other known bivalves is provided in Figure S2. *L. elliptica CHS* shows highest homology in its sequence to *Atrina rigida* (Japanese pearl oyster) and *Pinctada fucata* (stiff penshell oyster), with 72% identity at the nucleotide level and 82% and 80% identity over the deduced protein sequences, respectively (Table S1).

*C. Total RNA*

**Figure 3:**
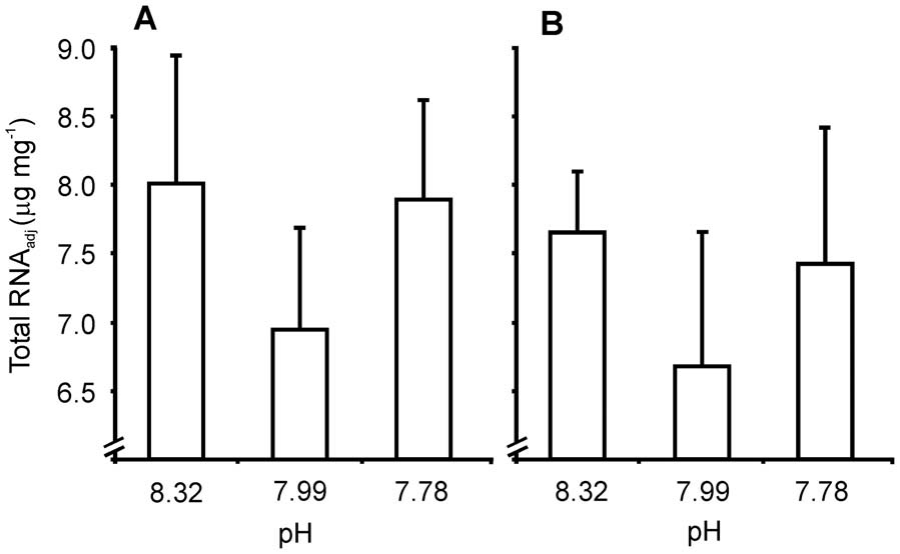
*Laternula elliptica* adductor tissue Total RNA_adj_ after A. 21 and B. 120 days at experimental pH.

Total RNA levels were lowest in *L. elliptica* from the Antarctic control pH treatment after both 21 and 120 days. Levels in the elevated and lowered pH treatments were higher, and similar to each other (Figure 3). This curvilinear response, similar to that noted for *HSP70*, was observed on each Day, although neither was statistically significant (Day 21: F = 0.40, p = 0.6815; Day 120: F = 0.37, p = 0.6975).

2. Oxygen consumption

**Figure 4:**
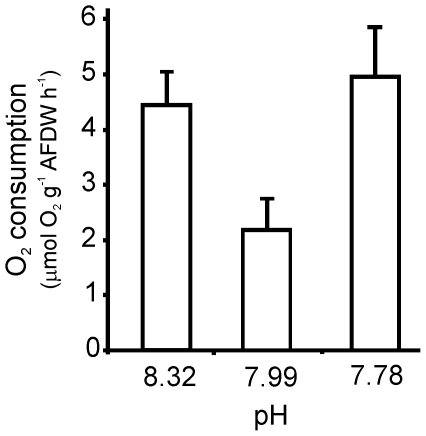
O_2_ consumption (µmol O_2_ g^−1^ AFDW h^−1^) of *Laternula elliptica* after 120 days at experimental pH.

O_2_ consumption rates were significantly higher in individuals from the elevated and lowered pH treatments compared with those from the Antarctic control at the end of the experiment (F = 5.53, p = 0.0159; Figure 4). There was no significant relationship between O_2_ consumption and *L. elliptica* SL (F = 1.17, p = 0.295, R^2^ = 0.067).

3. Physiological condition

**Figure 5:**
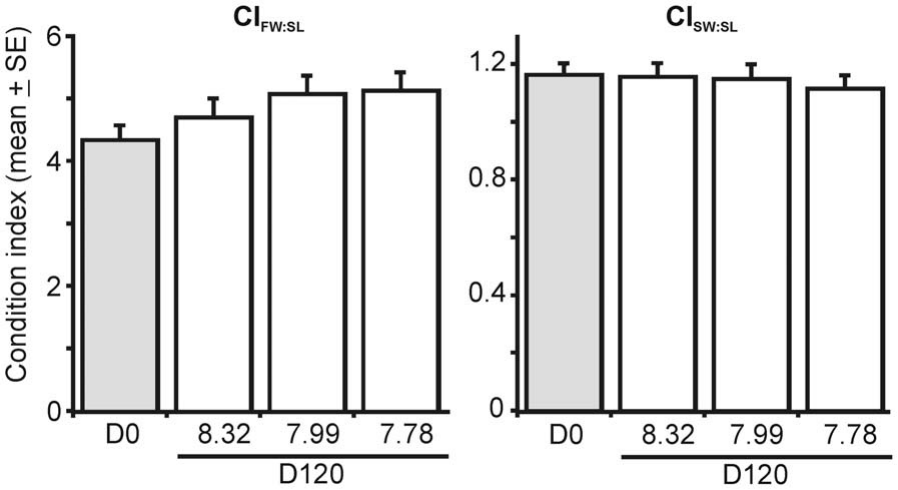
Physiological condition of *Laternula elliptica* in each experimental treatment on Day 120. Condition of individuals on Day 0 is also shown.

**Figure 6:**
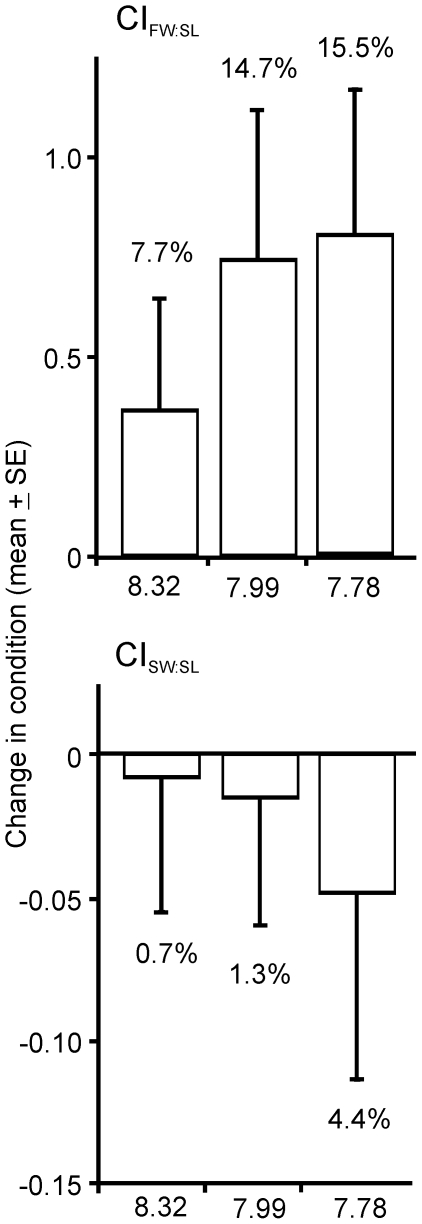
The change in each physiological condition index between Days 0 and 120. The % change that this represents is also given.

Physiological condition of *L. elliptica* as measured by CI_FW:SL_ on Day 120 did not differ between treatments, but overall condition had increased relative to Day 0 in all treatments, indicating the experimental conditions were favourable for maintenance and survival of the *L. elliptica* (Figure 5; 2-way ANOVA, Treatment: P = 0.3245; Day: P = 0.0443; Treatment*Day: P = 0.9434). While no significant difference between treatments on Day 120 was detected, the magnitude of average change in condition between the beginning and end of the experiment did not appear to be random (Figure 6, Pearson's R = −0.48, p = 0.0450, n = 18). The average increase in CI_FW:SL_ over the experiment was lowest at elevated pH (7.7%; Figure 6).

The CI_SW:SL_ index on Day 120 was not significantly different between treatments, nor was there any significant difference between Days 0 and 120 (Figure 5; Treatment: p = 0.9956; Day: p = 0.6332; Treatment*Day: p = 0.7730). Although Figure 6 suggests the magnitude of average change in CI_SW:SL_ between the beginning and end of the experiment may not be random, no significant correlation with pH was observed due to the high within-treatment variation, mainly due to the presence of a single high value in both the Antarctic control and low pH treatments (Figure 6, Pearson's R 0.29, p = 0.2655, n = 18). Preliminary examination of the outer surfaces of the *L. elliptica* shells did not reveal any obvious signs of degradation.

As there was no increase in shell length of the slow growing adult *L. elliptica* over the experiment, any change in CI_FW:SL_ or CI_SW:SL_ can be attributed to a change in flesh weight or shell weight, respectively.

## Discussion

Functioning of adult *Laternula elliptica* was affected by pH levels predicted for the Southern Ocean in coming decades (pH 7.78) after as little as 21 days exposure. Although no mortality occurred, *L. elliptica* in this lowered pH treatment were more stressed (Figure 1) and exhibited significantly higher basal metabolic rates (Figure 4) than *L. elliptica* from the Antarctic control (pH 7.99). Interestingly, this response was also noted for *L. elliptica* from the elevated pH treatment (pH 8.32), thus demonstrating a negative response by this bivalve to a change in pH in general. Most importantly, we noted a differential response of *L. elliptica* to lowered compared to elevated pH for *CHS* gene activity (as indicated by mRNA transcript abundance, Figure 2). CHS expression increased with decreasing pH, indicating an effect on the shell formation process of *L. elliptica* which appears specific to a decrease in pH. Total RNA content and *HSP70* gene expression level did not show a statistically significant response to our experimental treatments, although the pattern of both responses mirrored that of the basal metabolic rates (cf. Figures 1, 3 and 4). The *HSP70* response was close to being significant, with clear differences between individuals from the Antarctic control and both the elevated and lowered pH (Figure 1). The different response variables measured were influenced by pH in differing ways, indicating the importance of assessing a variety of different factors to determine the likely effect of pH change on organism functioning. While these effects on *L. elliptica* did not translate to statistically significant differences in either of the physiological condition indices we measured over the 120 day experiment (Figure 5), we suggest that the sustained cost of increased stress, metabolic and calcification rates may well affect *L. elliptica* condition (and thus growth and reproduction) in the longer term [47].

### Metabolism and growth

Reduced metabolism is a recognised strategy in Antarctic invertebrate fauna to minimise energy expended during routine ‘maintenance’ and thus have more energy available to invest in reproduction and growth [48]. In this experiment we have used oxygen consumption rates as a proxy for basal metabolism (e.g., [29], [49]). In less than optimal environmental conditions, we may expect to see basal metabolic rates increase as the organism works harder to maintain the status quo, and this may in turn result in changes in overall physiological condition. In our experiment oxygen consumption rates of *L. elliptica* in the Antarctic control were 2.2 µmol g^−1^ AFDW h^−1^, 2.3 and 2.1 times lower than in the lowered and elevated pH treatments, respectively. These results illustrate that while *L. elliptica* can function at pH's different from those they currently experience in Antarctica it is energetically more stressful for them to do so, indicating they are optimally adapted to their present environment. Metabolic rates (also measured as O_2_ consumption) in the bivalve *Yoldia eightsi* increased under higher temperatures [50], and a similar pattern has been noted for a range of other Antarctic marine invertebrates [51], [52]. Seasonal increases of 3 and 3.7 times between winter and summer have been reported for Antarctic Peninsula *L. elliptica* [53], [54], a likely response to changes in temperature and food availability. As no field measurements of oxygen consumption by McMurdo Sound (or indeed, Ross Sea) *L. elliptica* have been made, we are unable to comment on the magnitude of the differences between our experimental treatments relative to seasonal or spatial differences in Ross Sea *L. elliptica*. However, we do note that the O_2_ consumption rates in our Antarctic control animals were considerably lower than those recorded for *L. elliptica* from the Antarctic Peninsula region. [55] measured average rates of 73.3 and 49.6 µmol O_2_ h^−1^ 10.6 g^−1^ AFDW for *L. elliptica* from Rothera and Signy Island, respectively, at 0.0°C. When converted to enable direct comparison to our measurements, these rates are considerably higher than the average 2.2 µmol h^−1^ g^−1^ AFDW noted in the −1.8°C Antarctic control treatment of this experiment (i.e., Rothera 6.9, Signy 4.7 µmol h^−1^ g^−1^ AFDW), perhaps reflecting the influence of temperature on respiration. Furthermore, these differences in *L. elliptica* basal metabolism between Antarctic locations indicate that the functional response of this bivalve to ocean acidification may vary depending on their Antarctic environment.

Metabolic differences can also be reflected in short term growth rates as measured by total RNA (protein synthesis potential; [56]). Total RNA levels provide a measure of proteins synthesised during tissue growth as well as during physiological stress responses, and were relatively high in *L. elliptica* from the elevated and lowered pH treatments compared with the Antarctic control (Figure 3). Effects on total RNA levels were variable and, consequently, were not statistically significant on either Day 21 or Day 120 (Figure 3). We expect that they may have become significant given a longer experimental duration. Adductor muscle total RNA levels in our experiment were within the range of those found in *L. elliptica* collected at three other McMurdo Sound locations (i.e., Dunlop Island: 8.20±1.17 µg mg^−1^, n = 6; Spike Cape: 4.41±0.22 µg mg^−1^, n = 10; Cape Evans: 3.96±0.31 µg mg^−1^, n = 6; Joanna Norkko, unpublished data).

### Stress

An increase in protein synthesis (including production of heat shock proteins, HSPs) has also been noted in *L. elliptica* in response to another stressor, temperature [39], [40], [57]. HSPs in the normal cell state assist in the folding of native polypeptides, and their induction under stress conditions prevents production of cytotoxins and stabilises denatured proteins (e.g., [32], [58], [59]). HSPs can be produced in response to a large variety of environmental stressors (e.g., freshwater input in the intertidal [60]; osmotic stress [61]; presence of oxygen radicals and toxicants [62]), and we may expect a similar response to pH change. We have demonstrated a substantial up-regulation (increased production) of *HSP70* gene transcript levels in *L. elliptica* mantle tissue in response to pH levels that are both lowered and elevated relative to existing Antarctic conditions (Figure 1). This up-regulation is not unexpected as either pH directionality is likely to register as a stress to *L. elliptica*, stimulating induction of HSPs. HSP mRNA levels typically rapidly rise within hours following introduction to a stressor and generally reach their maxima following three hours of post-stress recovery [63], [64]. Our analysis shows that at 21 days following the initiation of the stress exposure (high or low pH), *HSP70* levels in these bivalves remain elevated relative to individuals from the Antarctic control. The magnitude of the *HSP70* up-regulation we observed is in line with experimental observations of the heat shock response (HSR [64]) in *L. elliptica* in response to the more classically studied stressor, temperature. *L. elliptica* constitutively express *HSP70* [39], a common phenomenon for Antarctic species, and further sustained up-regulation in response to pH as a stressor may in fact lead to deleterious effects on other cellular and organismal processes (e.g., [65]). There is an energetic cost associated with induction of the HSR and the HSR system in *L. elliptica* is likely to have evolved under strong trade-off constraints [59]. Thus, in this experiment, the increased respiration rates we observed at pHs either elevated or decreased relative to the Antarctic control may relate in part to the extra energy required to mount and maintain the HSR over an extended period of time.

### Shell (mineralogy and dissolution)

As *L. elliptica* shell is comprised purely of aragonite, one of the most soluble forms of CaCO_3_, it may be considered particularly susceptible to ocean acidification (e.g., [5], [66]). Aragonite was undersaturated in our lowered pH treatment (pH 7.78, Ω_Ar_ = 0.71) and the % change (loss) in CI_SW:SL_ after 120 days was the highest of all treatments (4.4%; Figure 6B, or 0.037% per day), although this correlation was not statistically significant. [9] reported a 2.767±0.607% loss in shell weight for *L. elliptica* shells held at 7.4 pH (Ω_Ar_ = 0.47) for 63 days, the equivalent of 0.044% per day. While a greater dissolution rate may be expected in the more undersaturated Ω_Ar_ conditions of the [9] experiment, we would urge caution in direct comparison of daily shell weight loss between experiments given that [9] studied empty shells and that dissolution may not be linear with time.

After just 21 days we found that lowering pH resulted in a significant increase in expression (up-regulation) of the *CHS* gene, which codes for a key enzyme involved in synthesis of bivalve shells [28], [67], thus indicating the animals were working harder to calcify. In contrast, elevated pH resulted in decreased *CHS* gene expression (down-regulation). Chitin is a major component of the bivalve shell [28], and forms the organic ‘framework’ within which CaCO_3_ minerals are subsequently deposited [67]. The enzymes involved in chitin synthesis, and chitin synthase in particular, are important not only in providing mechanical strength, but also in coordinating the shell formation and mineralisation process [67]. Inhibition of chitin synthesis has been clearly demonstrated to negatively affect survival and increase abnormal shell development rates in early *Mytilus galloprovincialis* larvae [67], [68]. In addition, larval shells formed under chitin synthesis-inhibited conditions were shown to be more soluble in distilled water [67]. Although *CHS* gene expression is not a direct measure of calcification, given that it encodes for an enzyme that is important in the shell mineralisation processes, effects on *CHS* gene expression could potentially affect the basic structure as well as the mineralisation, solubility and thus integrity of the shell. This is the first study examining changes in expression of *CHS* in response to ocean acidification (or other stressors) in any mollusc. The up- and down-regulation of *CHS* gene expression observed in our lowered and elevated pH treatments, respectively, is an important finding as it provides evidence for biological control over this process in response to changing environmental conditions, which may afford the organism a mechanism for some degree of acclimation or adaptation to future ocean acidification scenarios.

### Experimental vs Antarctic conditions

The conditions of our laboratory experiment were as close as possible to those of the Antarctic situation. Our pH and Ω_Ar_ measurements from Granite Harbour and New Harbour, and the pH reported for Cape Armitage by [23] show high agreement with the experimental values of our Antarctic control treatment (Table 1). The A_T_ of the seawater used in our experiment was naturally lower than that measured in the McMurdo Sound environment (by approximately 80 µmol kg^−1^; Table 1), although it was similar to that reported by [10] for cold, high latitude waters (2271±28.3). At -1.76°C, our experimental water temperatures were only slightly warmer than ambient spring water temperatures recorded for several locations in McMurdo Sound (−1.92°C; [69], [70]), and were very similar to those recorded in Terra Nova Bay where *L. elliptica* are also common (−1.8°C, authors' unpublished data). Salinities were also similar to those measured in McMurdo Sound in this study (Table 1), and by [70].

The experimental conditions were generally favourable for the maintenance and survival of *L. elliptica*, as shown by the increase in physiological condition CI_FW:SL_ over the 120 day experiment in all treatments (Figure 5). Because there was no measurable increase in SL of the adult *L. elliptica* over the experiment, this change was purely due to an increase in flesh weight. There was no significant difference in absolute CI_FW:SL_ between treatments on Day 120 (Figure 5), although the magnitude of this gain in condition between Day 0 and Day 120 increased significantly with decreasing pH (Figure 6). However, the significance of this was driven by the lower gain in condition of individuals in the elevated pH treatment, with little real difference between individuals from the lowered and Antarctic control pH treatments (Figure 6). For logistical reasons, *L. elliptica* were not held in sediments during these experiments, thus these experiments have not allowed for any buffering of the response to changing pH by burial. However, as pH within the sediment column is generally lower than the water column above [71], there will still be direct contact between the overlying seawater and the infaunal organism during feeding and respiration, and the CaCO_3_ content of the Granite Harbour sediments was very low, we do not anticipate that our results have overestimated the magnitude of the *L. elliptica* response.

In ocean acidification experiments, organisms are subjected to often-large pH changes which, of necessity, occur at a much faster rate than that predicted for their natural environments (i.e., years-decades). This has led to concern that these are in fact ‘shock-response’ experiments. It is also worth pointing out that in coastal and estuarine environments [72] and areas of oceanic upwelling [73], pH may change markedly over time scales more akin to those of experiments (i.e., hours to days). Of note are the high pCO_2_ levels of our McMurdo Sound water samples (410 and 440 µatm at Granite Harbour and New Harbour, respectively). The distance between these two coastal locations (ca. 90 km) and the fact that they were sampled in different years, indicates that the pCO_2_ levels of these high latitude coastal sites is already high in spring/early summer. Data are urgently needed to determine spatial and temporal variation in pCO_2_ and pH in Antarctic coastal regions, to put results of experiments into context of natural environmental conditions.

### Concluding comments

*L. elliptica* are a long lived species: their life span is estimated at 36 years [74], and they take about 20 years to reach 100 mm SL [26], [75]. The duration of this experiment is just a very small portion of their life span, and the effect on functioning of *L. elliptica* at a lowered pH of 7.78 is of concern given the predicted changes in Southern Ocean pH and aragonite saturation for the coming decades [19]. While many studies have focussed their investigations on juveniles due to the increased susceptibility of early life stages to environmental perturbations (e.g., [76]), examining effects on adults is also important, particularly for long lived Antarctic species which will experience this change in ocean chemistry within their generation.

We have shown significant effects on some crucial functions of *L. elliptica* at a pH only 0.2 units below current levels. Importantly, the observed changes in *L. elliptica CHS* gene expression provides evidence for biological control over the shell formation process, which may provide a mechanism for adaptation or acclimation to future changes in seawater carbonate conditions. We anticipate, however, that the energetic costs of maintaining these responses may have more serious implications for the condition of this key Antarctic bivalve in the longer term. In addition, increases in temperature predicted for Antarctic waters over the next 100 years, and associated changes in food supply (e.g., [77]), are likely to modify any effect of pH reductions alone on the behaviour and functioning of key benthic invertebrates. Future investigations should study synergistic effects, and should incorporate a range of response variables to build a more comprehensive picture of the likely ecological impacts of impending environmental change.

## Supporting Information

Table S1 **Designation of *CHS* gene family member status.**
(DOC)

Figure S1 **Nucleotide and deduced amino acid sequences of *Laternula elliptica* chitin synthase (*CHS*)** Numbered boxes refer to highly conserved regions found in many family 2 glycosyltransferases (GTF2) enzymes, including CHS. Regions 1 and 2, UDP-binding; region 1 is similar to the Walker A/P-loop motif and to the R-β-GKR consensus sequence of GTF2; region 2 is similar to the Walker B motif and to the K-β-DDGS consensus sequence of GTF2. Regions 3 and 4, donor saccharide-binding; region 3 is similar to the DXD motif; region 4 is similar to the G(X)_4_(Y/F)R consensus sequence important for enzyme processivity. Positions of the primers used in RT‐qPCR are highlighted on the cDNA sequence.
(DOC)

Figure S2 **Multiple alignment of deduced amino acid sequences of *Laternula elliptica CHS* with chitin synthases of other bivalves.** The *CHS* abbreviations, species and the Genbank accession numbers are as follows: LE*CHS*, *Laternula elliptica*, HQ186262; PF*CHS*, *Pinctada fucata*, AB290881; AR*CHS*, *Atrina rigida*, DQ081727; MG*CHS*, *Mytilus galloprovincialis*, EF535882. Bivalve sequences are numbered according to their complete Genbank entry whilst the amino acid sequence deduced from the LE*CHS* mRNA fragment is numbered 1‐318.
(DOC)
