# Supplementary material for: Ocean Acidification at High Latitudes: Potential Effects on Functioning of the Antarctic Bivalve Laternula elliptica
Source: PLoS One. 2011 Jan 5;6(1):e16069. doi: 10.1371/journal.pone.0016069 (PMC3016332; doi:10.1371/journal.pone.0016069)
Supplement: Table S1 — Designation of CHS gene family member status. (DOC) [file pone.0016069.s001.doc]

| **Closest database match**  **Accession: description** | **Score** | **Query coverage** | **% Identity** | **Probability** |
| --- | --- | --- | --- | --- |
| **A. Protein similarity** |  |  |  |  |
| AAY86556.1: *Atrina rigida* chitin synthase | 531 | 99% | 82% | 4e-149 |
| BAF73720.1: *Pinctada fucata* chitin synthase | 527 | 99% | 80% | 7e-148 |
| ABQ08059.1: *Mytilus galloprovincialis* chitin synthase | 512 | 99% | 77% | 3e-143 |
| **B. Nucleotide similarity** |  |  |  |  |
| AB290881.1: *Pinctada fucata* *PfCHS1* mRNA for chitin synthase, complete cds | 560 | 100% | 72% | 6e-156 |
| DQ081727.1: *Atrina rigida*, chitin synthase mRNA, complete cds | 545 | 100% | 72% | 1e-151 |
| EF535882.1: *Mytilus galloprovincialis* chitin synthase (*CHS1*) mRNA, complete cds | 365 | 99% | 68% | 3e-97 |
| EZ420589.1 TSA: *Pinctada maxima* PmaxCL475Contig1, mRNA sequence | 266 | 48% | 72% | 2e-67 |

Designations are based on BLAST match results from database sequence similarity search of A. protein databases (blastx), and B. nucleotide databases (blast) using nucleotide query. Common names are as follows: *Atrina rigida*, Japanese pearl oyster; *Pinctada fucata*, stiff penshell; *Mytilus galloprovincialis*, Mediterranean mussel; *Pinctada maxima*, pearl oyster/gold lip oyster/white lip oyster.
