# Supplementary material for: Ocean Acidification at High Latitudes: Potential Effects on Functioning of the Antarctic Bivalve Laternula elliptica
Source: PLoS One. 2011 Jan 5;6(1):e16069. doi: 10.1371/journal.pone.0016069 (PMC3016332; doi:10.1371/journal.pone.0016069)
Supplement: Figure S1 — Nucleotide and deduced amino acid sequences of Laternula elliptica chitin synthase (CHS) Numbered boxes refer to highly conserved regions found in many family 2 glycosyltransferases (GTF2) enzymes, including CHS. Regions 1 and 2, UDP-binding; region 1 is similar to the Walker A/P-loop motif and to the R-β-GKR consensus sequence of GTF2; region 2 is similar to the Walker B motif and to the K-β-DDGS consensus sequence of GTF2. Regions 3 and 4, donor saccharide-binding; region 3 is similar to the DXD motif; region 4 is similar to the G(X)4(Y/F)R consensus sequence important for enzyme processivity. Positions of the primers used in RT‐qPCR are highlighted on the cDNA sequence. (DOC) [file pone.0016069.s002.doc]

1 caggtgtacgcgtgcgcaacaatgtggcacgagacccgtcaagaaatgacycagttattg

Q V Y A C A T M W H E T R Q E M X Q L L

61 argtctttgttcagattggactacgttcattgcgcaagtcgtctggcccaggaaaagttt

X S L F R L D Y V H C A S R L A Q E K F

121 cgaataaaggacccggattactatgacctggaaattcacgtcatttttgatgacgctatg

R I K D P D Y Y D L E I H V I F D D A M

181 gagttaaatgacgacgtggacaaatatgtacccaacatgttcgtcaaacagctcatcgat

E L N D D V D K Y V P N M F V K Q L I D

241 tgtatggaggacgccgccagatccgtggtaaagggaccaattatgatgtccgctcctatc

C M E D A A R S V V K G P I M M S A P I

301 aaaaccagcaccccttacggcggtcgcttgacctggacaatgccgggtcgaaccaagatg

K T S T P Y G G R L T W T M P G R T K M

361 gtggttcacgtcaaggacaagaacaagattcgtcacagaaagagatggtctcagtgcatg

V V H V K D K N K I R H R K R**^1^** W S Q C M

421 tatctgtactatctgctgggttacaaactcttcgggaccaaggaaggagataaggccttt

Y L Y Y L L G Y K L F G T K E G D K A F

481 actgaggacctctcggagatggacagcaaggtgtccaaggcaaggaacaggaagaagggc

T E D L S E M D S K V S K A R N R K K G

541 agatcaaagaaggacaacctctctcgaccaatcaaatctctcttcaaccgaatggacacg

R S K K D N L S R P I K S L F N R M D T

601 gaacaatacgaacaggctgagaacacattcatcttgacactggacggtgacgtcgacttc

E Q Y E Q A E N T F I L T L D G**^2^** D V D**^3^** F

661 aggccagaatctgtcaagcttctgattgataggatgaagaagaaccggaaggtcggtgcc

R P E S V K L L I D R M K K N R K V G A

721 gtctgcggcagaattcacccgattggatcaggtcctatggtttggtaccaacagttcgag

V C G R I H P I G S G P M V W Y Q Q F E

781 tacgchgtyggccattggctkcaraaagcggcagagcacgtgtttggatgtgtcctctgt

Y X X G H W X X K A A E H V F G C V L C

841 tgtcctgggtgtttctcgctgttccgaggctccgccctcatggacgacaacgttatcaag

C P G C F S L F R**^4^** G S A L M D D N V I K

901 atgtacacaacgaaaccgacagaggcacggcattacatccagttcgaacaagg

M Y T T K P T E A R H Y I Q F E Q
