# Supplementary material for: Ocean Acidification at High Latitudes: Potential Effects on Functioning of the Antarctic Bivalve Laternula elliptica
Source: PLoS One. 2011 Jan 5;6(1):e16069. doi: 10.1371/journal.pone.0016069 (PMC3016332; doi:10.1371/journal.pone.0016069)
Supplement: Figure S2 — Multiple alignment of deduced amino acid sequences of Laternula elliptica CHS with chitin synthases of other bivalves. The CHS abbreviations, species and the Genbank accession numbers are as follows: LECHS, Laternula elliptica, HQ186262; PFCHS, Pinctada fucata, AB290881; ARCHS, Atrina rigida, DQ081727; MGCHS, Mytilus galloprovincialis, EF535882. Bivalve sequences are numbered according to their complete Genbank entry whilst the amino acid sequence deduced from the LECHS mRNA fragment is numbered 1‐318. (DOC) [file pone.0016069.s003.doc]

LE*CHS* QVYACATMWHETRQEMTQLLXSLFRLDYVHCASRLAQEKFRIKDPDYYDLEIHVIFDDAM 60

PF*CHS* ...V................K............K........N...FFN..L.......F 1391

AR*CHS* ....................K.....................H........L.I.....F 1392

MG*CHS* F..V................K............K...D........FF...M.I.....F 1397

LE*CHS* ELNDDVDKYVPNMFVKQLIDCMEDAARSVVKGPIMMSAPIKTSTPYGGRLTWTMPGRTKM 120

PF*CHS* ..DEK....I..S.....VE..............SLLP.E.VA.....K.I.....H..L 1451

AR*CHS* ..D.K.......S..R...E..............SIQP.E.IP.......V.....H..L 1452

MG*CHS* ..DES....I..G..RL.Y..............VIL.S.E.VP.....K.I.....H..L 1457

LE*CHS* VVHVKDKNKIRHRKRWSQCMYLYYLLGYKLFGTKEGDKAFTEDLSEMDSKVSK-ARNRKK 179

PF*CHS* ...M.....M...........M..........A..A.NYMM..A---E.SMT.-LK.... 1507

AR*CHS* N..M.....M......................AR.A.RYMA..A---E.SMT.-VK.... 1508

MG*CHS* H..M.....M........V..M..........AY.A..FMM.EMDK-ENPM..NV.Q... 1516

LE*CHS* GRSKKDNLSRPIKSLFNRMDTEQYEQAENTFILTLDGDVDFRPESVKLLIDRMKKNRKVG 239

PF*CHS* .K...TQR...LR...M..TP......D.............K.D............K... 1567

AR*CHS* SK...CQR...LR...M..TPD.........M...........D............K... 1568

MG*CHS* NKG..KEK...L....S..NA...D......L.........K.DA............... 1576

LE*CHS* AVCGRIHPIGSGPMVWYQQFEYAVGHWLQKAAEHVFGCVLCCPGCFSLFRGSALMDDNVI 299

PF*CHS* .....................................................V.....M 1627

AR*CHS* .....................................................I.....L 1628

MG*CHS* ..................E..................................V.....L 1636

LE*CHS* KMYTTKPTEARHYIQFEQG 318

PF*CHS* ................... 1646

AR*CHS* ................... 1647

MG*CHS* .....P............. 1655
